# Unravelling the Role of Epigenetic Modifications in Development and Reproduction of Angiosperms: A Critical Appraisal

**DOI:** 10.3389/fgene.2022.819941

**Published:** 2022-05-18

**Authors:** Priyanka Kumari, Sajid Khan, Ishfaq Ahmad Wani, Renu Gupta, Susheel Verma, Pravej Alam, Abdullah Alaklabi

**Affiliations:** ^1^ Conservation and Molecular Biology Lab., Department of Botany, Baba Ghulam Shah Badshah University, Rajouri, India; ^2^ Division of Soil Sciences & Agricultural Chemistry, Faculty of Agriculture Sher e Kashmir University of Agricultural Sciences and Technology, Chatha, India; ^3^ Department of Botany, University of Jammu, Jammu, India; ^4^ Department of Biology, College of Science and Humanities, Prince Sattam bin Abdulaziz University (PSAU), Alkharj, Saudi Arabia; ^5^ Department of Biology, College of Science, University of Bisha, Bisha, Saudi Arabia

**Keywords:** epigenetic modification, DNA methylation, histone proteins, stress response, plant hormones, transposon silencing, miRNA

## Abstract

Epigenetics are the heritable changes in gene expression patterns which occur without altering DNA sequence. These changes are reversible and do not change the sequence of the DNA but can alter the way in which the DNA sequences are read. Epigenetic modifications are induced by DNA methylation, histone modification, and RNA-mediated mechanisms which alter the gene expression, primarily at the transcriptional level. Such alterations do control genome activity through transcriptional silencing of transposable elements thereby contributing toward genome stability. Plants being sessile in nature are highly susceptible to the extremes of changing environmental conditions. This increases the likelihood of epigenetic modifications within the composite network of genes that affect the developmental changes of a plant species. Genetic and epigenetic reprogramming enhances the growth and development, imparts phenotypic plasticity, and also ensures flowering under stress conditions without changing the genotype for several generations. Epigenetic modifications hold an immense significance during the development of male and female gametophytes, fertilization, embryogenesis, fruit formation, and seed germination. In this review, we focus on the mechanism of epigenetic modifications and their dynamic role in maintaining the genomic integrity during plant development and reproduction.

## Introduction

Epigenetics is the study of mitotically and/or meiotically heritable changes in gene function that cannot be explained by changes in the DNA sequence (Waddington 1957; Waddington, 2012; Iwasaki and Paszkowski, 2014). Epigenetic changes are induced through behavioral or environmental factors that may affect the way the genes work (Niederhuth and Schmitz, 2017; Parker et al., 2021). These modifications are reversible and do not change the sequences of DNA but alters the way in which DNA sequences are read. Epigenetic modifications include DNA methylation (Bouyer et al., 2017), histone modifications (Zhou, 2009; Liu et al., 2010), ubiquitination of histone N-tails, and posttranscriptional silencing through small noncoding RNAs and RNA-mediated mechanisms (Slotkin and Martienssen, 2007; Matzke and Mosher, 2014). Gene and transposon activity get affected by epigenetic changes in the DNA present within a chromatin (Lippman et al., 2004; Rodrigues and Zilberman, 2015). Epigenetics regulate flowering time in plants through transposon silencing, paramutation, and genomic imprinting (Yaish et al., 2011; Ay et al., 2014; Blüumel et al., 2015; Parker et al., 2021). Epigenetic changes are inherited through alleles or epialleles. Epialleles are the sites that get transmitted to the next generations after being retained stably in the chromatin state. Epialleles act as supplementary sources of variation to regulate phenotypic diversity. In plant species, epialleles affect floral morphology, time of flowering, resistance against diseases, and leaf senescence (Brukhin and Albertini, 2021). The present review is a summary of the information available on different epigenetic modifications that appear to be important in growth, development, and reproduction of plants.

### Epigenetic Modifications in Plants

Plants being sessile in nature are invariably affected by changing environmental conditions. However, they have the ability to adapt their biological processes according to the changing environments. They interact with their environment through consistent adjustments at the molecular level by modifying the patterns of gene expression (Yaish et al., 2011). Epigenetic regulations assist plants in increased tolerance against different environmental stresses by reprogramming their developmental stages, such as flowering time (Barozai and Aziz, 2018). In transgenic plants, epigenetics helps to understand the problems related to suitable expression of newly introduced transgenic segments (Madhusudhan, 2015). Epigenetic changes are conserved in plants and influence the structure of the chromatin which in turn regulates the gene expression. Epigenetic mechanisms are important to regulate various biological processes and disruption of any one of the epigenetic mechanisms leads to developmental abnormalities in plants. Therefore, epigenetic changes play dynamic roles in the growth and development of plants (Zhang et al., 2018).

Different biological pathways such as phytohormone signaling, photoperiodism, and vernalization in combination with the environmental signals (epigenetic changes) regulate flowering time by integrating internal state of development of plants (An et al., 2004; He, 2009; Amasino, 2010; Andrés and Coupland, 2012; Sun et al., 2014; Burgarella et al., 2016). This network of flowering regulation involves FLOWERING LOCUS D (FLD) and FLOWERING LOCUS C (FLC) gene transcription which are controlled through epigenetic mechanisms such as ubiquitination, acetylation/deacetylation, and methylation/demethylation concealed by hormone signaling. HISTONE DEACETYLASE 6 (HDA6) protein increases the rate of expression of FLC gene but ethylene sets off HDA6 expression. FLC is suppressed by FLOWERING LOCUS D through demethylation of H3K4me2 which facilitates H4 deacetylation in the same locus. H3K27me3 is added by PRC2 marking into FLD chromatin. This also engages PICKLE to link with DELLA which in turn facilitates the repression of FLC (Bastow et al., 2004; De Lucia et al., 2008; Jiang et al., 2008; Choi et al., 2009).

### Regulation of Epigenetic Modifications

Phenotypic plasticity within the plant species plays an important role in adaptation to different environmental conditions allowing different cultivars to adjust and grow. Plasticity maintains the homeostasis within changing environmental conditions that allows better gene expression to adapt to different biotic or abiotic constrains (disease, herbivory, plant–plant competition, altitude, soil type, seasonal, day length, rain, and ambient temperature) (Gratani, 2014). Genetic plasticity within the plants is inferred by different epigenetic modifications that are regulated by DNA methylation, histone modification, transposon modification, noncoding RNAs, and chromatin modulation (Zhang et al., 2018; Zhang et al., 2008; Zhang et al., 2013).

### DNA methylation

DNA methylation is a chromatin modification in plants and is conceivably inherited mitotically or meiotically over generations. DNA methylation is catalyzed by cytosine methyltransferases. It involves the addition of a CH_3_ group (methyl group) at the fifth carbon position on cytosine residue generating 5-methyl cytosine in a sequence-specific manner. The methyl group acts as a platform for various protein complexes to attach and modify the chromatin scaffolds causing altered gene expression (Niederhuth and Schmitz, 2017). On the basis of the target sequence, methylation is of two types: asymmetrical and symmetrical methylation. Symmetrical methylation is CG and CHG methylation and asymmetrical methylation is CHH methylation (where H denotes any nucleotide other than guanine). Both symmetric, i.e., CG and CHG, and asymmetric methylation, i.e., CHH, exist in plants (Jacobsen and Meyerowitz, 1997). Only some genes are methylated in plants within a gene body, and methylation is restricted only to CG sites (Niederhuth and Schmitz, 2017). DNA methylation is found to be higher at repetitive sequences than genic regions in case of plant species. To maintain genome stability, silencing of TEs is important which can be mediated through RNA-directed DNA methylation (RdDM) (Slotkin and Martienssen, 2007; Matzke and Mosher, 2014). Despite having a pivotal role in different biological processes, DNA methylation applications in crop improvement are not fully investigated.

**FIGURE 1:**
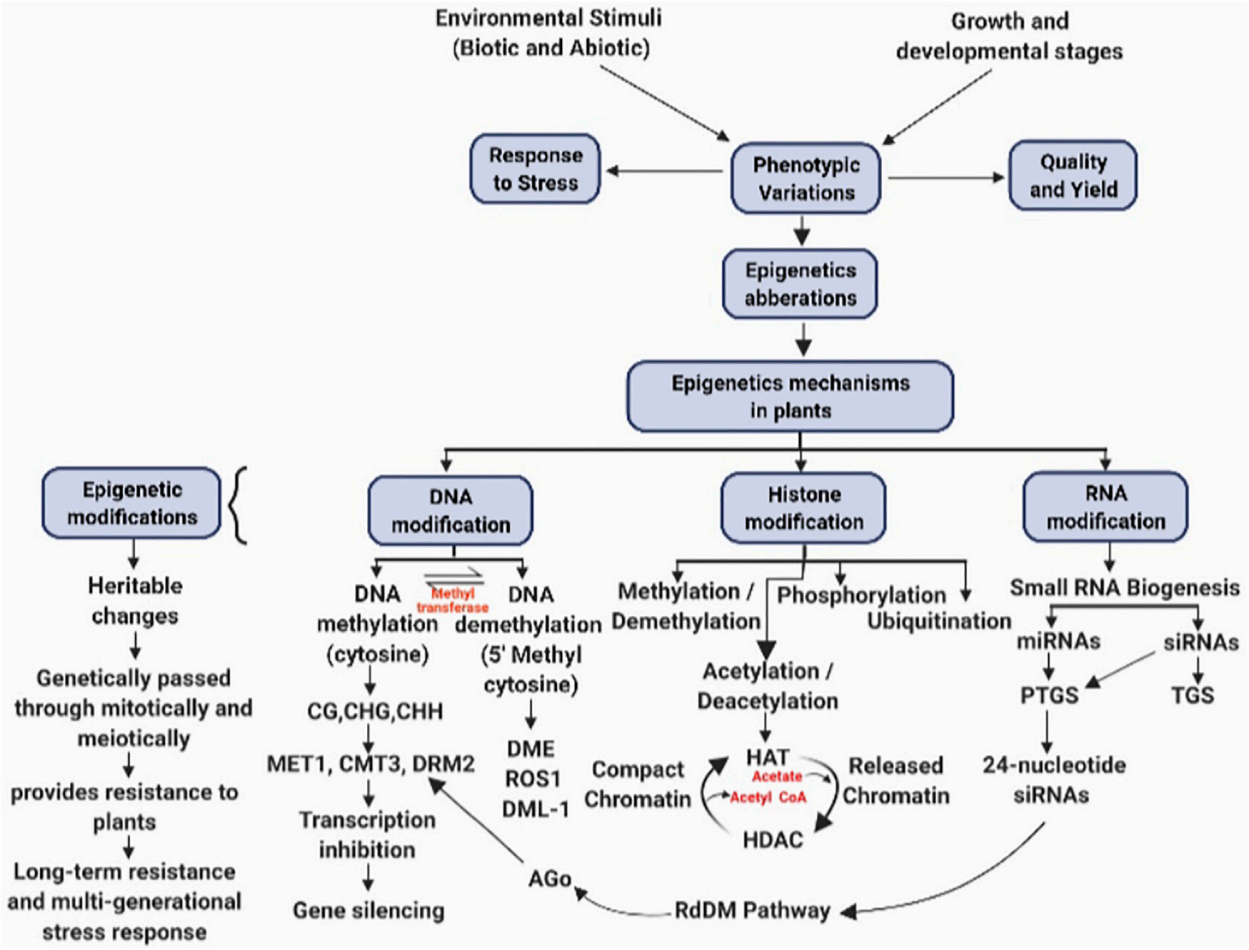
Epigenetic changes in response to stress management during growth and development of plant.

DNA methylation is induced biochemically as an epigenetic heritable change initiated through enzymes. It entails a shift of a methyl group to the fifth position on the cytosine residue and is catalyzed by DNA methyltransferases utilizing S-adenosylmethionine (Thapa and Shrestha, 2020). DNA methylation occurs at cytosine regions, *viz*., CG, CHG, and CHH (H stands in for A, T, or C) (Figure 1). METHYLTRANSFERASE 1 (MET1) enzyme catalyzes the methylation of CG. After the completion of DNA replication process, MET1 identifies hemi-methylated CG dinucleotides and methylates the unchanged cytosine in the daughter strand (Kankel et al., 2003; He et al., 2011). DNA methyltransferases CHROMOMETHYLASE 3 (CMT3) and CHROMOMETHYLASE 2 (CMT2) are said to catalyze CHG methylation (Lindroth et al., 2001; Stroud et al., 2014). Depending on the chromosomal region, CHH methylation is catalyzed through DOMAINS REARRANGED methyltransferase 2 (DRM2) or CMT2 methyltransferases. Methylation through CHROMOMETHYLASE 2 is catalyzed at histone H1–containing heterochromatin sites, while DRM2 catalyzes methylation at RdDM target areas (Zemach et al., 2013; Zhang et al., 2018) (Figure 1). There are two types of DNA methylation mechanisms: active and passive DNA methylation. A particular protein participates in the active process and demethylates the DNA sequence. The base excision repair pathway is involved. During DNA replication, methylation of cytosine is replaced with unmodified cytosine in a passive process. The reduction of activity of DNA methylases such as METHYLTRANSFERASE 1 (MET1) and CHROMOMETHYLASE 3 (CMT3) causes the addition of non-modified cytosine during DNA replication (Ibarra et al., 2012). In the case of plants, cytosine methylation has been extensively researched (Ruffini Castiglione et al., 2002). Cytosine alterations are not constant and vary greatly depending on the stages of development in plants and environmental factors (Burn et al., 1993).

DNA methylation is recruited *de novo via* DRM2 at all sequence contexts, and its role in CHH methylation is more prominent because CHH methylation depends only on *de novo* methylation. The RdDM pathway regulates the DRM2 activity. The RdDM pathway contains two plant-specific DNA-dependent RNA polymerases. The large subunit of these polymerases consists of proteins, i.e., NRPD1 and NRPE1, and functions specifically in transcriptional gene silencing and *de novo* DNA methylation. Single-stranded RNAs are produced through DNA-dependent RNA polymerase IV (Pol IV) in DNA repeat sites and in transposon regions. Pol IV transcription is facilitated by chromatin remodeling protein CLASSYs (CLSYs; CLSY1-4) (Yang et al., 2018; Zhou et al., 2018). Single-stranded RNA is converted to double-stranded RNAs *via* RNA-dependent RNA polymerase (RDR2). Double-stranded RNA is then cleaved by Dicer-like protein (DCL3) into 24-nt siRNA. This 24-nt siRNA binds to ARGONAUTE proteins (AGO4, AGO6, and AGO9). Loading of siRNA to ARGONAUTE proteins require KOW CONTAINING TRANSCRIPTION FACTOR 1 (KTF1)/SPT5-like protein. KTF1 (RNA-binding protein) binds to noncoding RNA transcripts produced from Pol V forming RNA-directed DNA methylation effector complex. Pol V generates single-stranded RNA transcripts in intergenic noncoding (IGN) regions. RNA transcripts generation *via* Pol V requires DRD1, DMS3, RDM1 and RDM4. RDM1 binds to single-stranded methylated DNA and leads to the recruitment of Pol V to chromatin regions. To catalyze new DNA methylation, *de novo* DNA methyltransferases DRM2 are directed to specific chromatin regions *via* effector complex (He et al., 2011; Xie et al., 2012). It has been reported that six homologous proteins act in a redundant way in the RdDM pathway. These proteins are found in *Arabidopsis* and named as FACTOR of DNA METHYLATION 1–5 (FDM1-5) and INVOLVED IN DE NOVO (IDN2/RDM12). They belong to the SGS3-like plant-specific protein family, and their rice homolog is X1. They have an important role in transcriptional gene silencing like the SGS3 protein family (Xie et al., 2012).

### Histone Protein Modifications

Histone modifications comprise an interesting part in epigenetics (Pfluger and Wagner, 2007). Histone proteins act as winder around which the segment of DNA gets wrapped and leads to the formation of a structural unit called as nucleosome. Nucleosomes comprise histone octamers consisting of two copies of each of the H2A, H2B, H3, and H4 histone proteins. The N-terminal tail of these histone proteins undergoes different modifications such as acetylation, methylation, sumoylation, ubiquitination, and phosphorylation. These histone modifications are correlated with either gene activation or repression. Several histone variants and enzymes are present in plants that modify histones posttranslationally and regulate gene expression (Zhou, 2009; Liu et al., 2010). Gene expression is brought about by the process of acetylation and phosphorylation, whereas gene expression is reduced through sumoylation and biotinylation. In the case of plants, deacetylation and methylation of H3K27 and H3K9 repress genes, while H3K4 and H3K36 acetylation and methylation activate gene expression. Epigenetic modifications participate in several biological processes like transcription, replication, and DNA repair by recruiting specific proteins needed in such processes (Jiang et al., 2009; Iwasaki and Paszkowski, 2014). Epigenetic modifications not only consist histone marks/modifications but also consist replacement with histone variants having different properties to influence gene expression (Liu et al., 2010). Chromatin immunoprecipitation applications following deep sequencing provide an insight for the genome-wide association studies regarding variants of histones and their posttranscriptional modifications (Butterbrodt et al., 2006). Responding to various biotic and abiotic stresses, histone modifications regulate the DNA transcription by interfering with the packaging structure either by activating the DNA to transcribe or making condensed structures thereby deactivating transcription machinery.

N-terminal tails of histones are the sites where most of the histone modifications take place. These modifications specify the function of chromatin and transcriptional activities (Jenuwein and Allis, 2001; Zhao et al., 2019). Histone modifications include methylation, acetylation, ubiquitination, and phosphorylation and occur at lysine and arginine residues. Histone methyltransferases (writers) are a group of SET domain which catalyzes histone lysine methylation, and downstream events are mediated by proteins (readers) which recognize specific lysine methylation. Two histone demethylases, i.e., Jumonji C (Jmj C) and lysine-specific demethylase 1 (LSD 1) catalyze the removal of histone lysine methylation (Zhao et al., 2019). In eukaryotes, the involvement of histone acetylation and methylation in gene expression regulation was first identified by Allfrey et al. (1964). It has been demonstrated that increase in histone acetylation leads to poor separation of sister chromatids in human fibroblasts and causes chromosomal defects during cell cycle in tobacco. Trichostatin A (TSA) is identified to have negative pleiotropic effects and has been found to decrease global histone deacetylation, resulting in an increase in acetylated histones (Cimini et al., 2003; Li et al., 2005).

The overexpression of antisense of the histone deacetylase gene (AtHD1) induces histone acetylation activity in *Arabidopsis thaliana*. The AtHD1 gene gives rise to pleiotropic phenotypes having a variety of developmental defects such as the suppression of apical dominance, ectopic expression of silenced genes, floral structure abnormalities, male and female sterility, heterochronic shift toward juvenility (Tian and Chen, 2001). In plant genome, the repressive state of heterochromatic regions is marked by H3K9me1 and H3K9me2. Heterochromatic regions are enriched with transposable elements and repetitive sequences. Heterochromatic regions in *Arabidopsis* are enriched with H3K27me1, and the association of H3K27me1 is catalyzed by plant-specific histone methyltransferases ARABIDOPSIS TRITHORAX-RELATED PROTEINS, i.e., ATXR 5 and ATXR 6. Mutation in H3K27me1 results in de-condensation of heterochromatin and the release of transposable silencing (Zhao et al., 2019). H3K27me3 deposition on chromatin is catalyzed by polycomb repressive complex 2 (PRC2) *via* histone methyltransferases. The subunits of PRC2 were first identified in *Drosophila melanogaster* (Shen et al., 2021). In plants, PRC2 deposits the H3K27me3 methylation and plays an important role in growth and developmental phases of plants. This modification is found in protein-coding genes and is regulated dynamically during the growth stages of plants. PRC2 consists of four components, *viz*., histone methyltransferases enhancer of zeste [E(z)], extra sex combs (Esc), suppressor of zeste 12 [Su(z)12], and the histone-binding nucleosome-remodeling factor 55 kDa (Nurf55, also called p55). One component of PRC2, i.e., E(z), specifically belongs to the SET [Su(var)3-9; E(z); trithorax] domain family and is responsible for histone H3 tri-methylation at Lys27 (Czermin et al., 2002; Butenko and Ohad, 2011; Simon and Kingston, 2013; Shen et al., 2021). In case of *Arabidopsis*, PRC2 components have multiple duplications, and there exists three homologs of E(z), *viz*., CURLY LEAF (CLF), SWINGER (SWN), and MEDEA (MEA); three homologs of Su(z), *viz*., EMBRYONIC FLOWER 2 (EMF2), VERNALIZATION 2 (VRN2), and FERTILIZATION-INDEPENDENT SEED 2 (FIS2); and one homolog of Esc, *viz*., COPY SUPPRESSOR OF IRA 1–5 (MSI1-5). FIS2 regulates mega-gametogenesis and endosperm development in plants during postfertilization events and EMF, and VRN polycomb repressive complexes regulate the development of sporophyte and phase transition, i.e., vegetative to reproductive in plants (Shen et al., 2021). Histone demethylases found in *Arabidopsis*, i.e., JUMONJI 13 (JMJ13), JUMONJI 30 (JMJ30), JUMONJI 32 (JMJ32), EARLY FLOWERING 6 (ELF6), and RELATIVE OF EARLY FLOWERING 6 (REF6), demethylate H3K27 and depress genes temporally or spatially for processes like flowering, signaling of hormones, and circadian clock control (Sanchez et al., 2020).

Histone methylation is influenced by environmental factors (Boyko and Kovalchuk, 2008; Kim et al., 2010). Global gene expression analysis and chromatin immunoprecipitation (ChIP) tests have revealed that histone H3 Lys4 methylation (H3K4) patterns in *Arabidopsis* respond dynamically to dehydration stress (van Dijk et al., 2010). The floral initiator SHK1 kinase BINDING PROTEIN 1 (SKB1) mutant line *skb1* provides an example of the interaction between environmental stress and blooming. SKB1 attaches to chromatin and raises the quantity of histone 4 Arg3 (H4R3) symmetric dimethylation (H4R3sme2) and causes FLC expression and a number of stress-responsive genes to be downregulated. As a result, its mutant characteristics include salt hypersensitivity, late flowering, and stunted development (Zhang et al., 2011; Chen et al., 2013; Cheng et al., 2019). The standard ABC model determines flower architecture at the molecular level (Bowman et al., 1991; Bowman et al., 2012). The geographical bounds of each floral whorl are determined by precise union of gene expression and protein interactions in this model (sepals, petals, stamens, and carpel). The A class gene APETALA2 (AP2) regulates target gene expression as part of a complex that it forms with TOPLESS (TPL) and HISTONE DEACETYLASE 19 in *Arabidopsis* (HDA19). AGAMOUS (AG) and SEPALATA3 (SEP3), the C class and E class genes, respectively, are negatively regulated by the transcription repressor complex. Deacetylation of H4K16 in regulatory areas of AG and SEP3 mediates gene suppression (Krogan et al., 2012). Expression studies revealed and identified additional HDACs expressed in reproductive tissues in *Arabidopsis*, i.e., HDA5, HDA6, HDA7, HDA9, HDA15, and HDA18. Their function in fruit or flower development is unknown. Only the function of HDA6 has been reported, and it plays a role in the regulation of blooming time. Histone H3K4 demethylase, i.e., FLOWERING LOCUS D (FLD), interacts directly with HDA6. The complex represses the expression of three flowering repressors: FLD, MADS AFFECTING FLOWERING 4 (MAF4), and MAF5 by removing the acetyl and methyl groups from histone 3 at their loci (Yu et al., 2011). HDA6 is one of the HDACs engaged in RdDM. RdDM is a plant-specific epigenetic process and small interfering RNA (siRNA)–mediated epigenetic mechanism which regulates the chromatin silencing of developmental genes, transposable elements, and repetitive elements. The RdDM mechanism involves a large number of participants whose actions may be broken down into a few simple phases (Matzke and Mosher, 2014). The RdDM machinery involves two kinds of transcripts, *viz*., Pol IV and Pol V transcripts. Pol IV transcribes long noncoding RNAs (lncRNAs), and the lncRNAs gets transformed to double-stranded RNAs (dsRNAs) through RDR2 (Haag et al., 2012). dsRNAs then gets converted into siRNAs by DICER-like 3 (DCL3). The siRNAs are loaded into AGO4 and reimported into the nucleus after being exported to the cytoplasm. siRNA direct AGO4 to nascent scaffold transcripts of Pol V through precise base pairing. siRNA, AGO4, and lncRNA scaffold derived from Pol V recruit histone deacetylases (HDACs) and DNA methyltransferases, which in turn silence the genomic loci transcribed by Pol V by the process of histone deacetylation and DNA methylation. Histone deacetylation characterizes RdDM-silenced promoters, which is mediated by RPD3-type histone deacetylase AtHDA6 in *Arabidopsis*, which is homologous to SIHDA3 of tomato. Deacetylation is essential for subsequent methylation by histone methyltransferases (HMTs), and to control siRNA-dependent heterochromatin, there is a requirement of functional AtHDA6 (Li et al., 2005; Aufsatz et al., 2007). The mutants of AtHDA6 display the revival of RdDM-silenced promoters in spite of the presence of an RNA-silencing signal. Reduced cytosine methylation indicates that AtHDA6 plays an important role in methylation maintenance. The physical connection of AtHDA6 with DMTs, MET1, and CMT3 may facilitate this function. Acetylases and deacetylases of histones have an important role in flowering and fruit development of tomato plant (Aufsatz et al., 2002). Cigliano et al. (2013) identified potential histone modifiers of AU4 tomato genome using RNA sequencing data of tomato genome (Tomato Genome C 2012) generated by worldwide collaboration sequencing. Also, they analyzed the expression profiles of each histone modification in the sample tissues used by using RNA sequencing data from the same source. Two histone acetylases S1HAG18 and S1HAG6 showed peak expression in the floral samples used, which indicates their function in reproductive development of tomato plant. Recently, 15 histone deacetylases have been discovered in tomatoes. SlHDA3 was found to be the tomato homolog of AtHDA6 which is expressed in all tissues having the highest blossom stage expression (Zhao et al., 2014). Another tomato homolog of AtHDA19, i.e., S1HDA1, was found to be significantly expressed in the flowering stage, and its expression was repressed at the fruiting stage. In yeast two-hybrid tests, it has been found that histone deacetylases, including S1HDA1, S1HDA3, and S1HDA4, interact with MADS-box transcription factors, i.e., TOMATO AGAMOUS1 (TAG1) and TOMATO MADS-BOX (TM29) (Zhao et al., 2014). Transcription factor TAG1 is required for the expression of both ethylene-dependent and ethylene-independent ripening genes (Klee and Giovannoni, 2011). TM29 is a homolog of SEPALLATA, which when silenced, leads to the formation of aberrant flowers and parthenocarpic fruits (Ampomah-Dwamena et al., 2002).

### Transposon Modifications

Most species have transposon elements (TEs) in their chromosomes, and multicellular eukaryotes have TEs as a key component of their genome. The majority of transposable elements are silenced epigenetically, although certain transposable elements have active transcription in epigenetic regulation mutants. Furthermore, environmental stress can trigger TE transcription, a mechanism that occurs across the evolutionary spectrum from bacteria to mammals (Capy et al., 2000). McClintock (1984) was the first to report that stress might cause TEs to shift, a result that has been widely corroborated in subsequent research (Grandbastien, 1998). Tnt1 and Tto1 are LTR-type Class I retroelements in tobacco, whose transposition is triggered by injury or through pathogen attack (Takeda et al., 2001; Perez-Hormaeche et al., 2008). Also, Bs1 LTR-type Class I retroelement in maize was found to transpose after viral infection (Johns et al., 1985). In *Arabidopsis*, heat stress induces transcription of ONSEN (LTR-type Class I retroelement), and it transposes into siRNA-defective mutants (Ito et al., 2011). LTR of ONSEN has a heat-responsive region that is activated by transcriptional heat stress responses (Cavrak et al., 2014). As a result, genes near or containing newly inserted ONSEN copies become heat-responsive (Ito et al., 2011). All the above examples of transposons are of Class I DNA transposons, and these transpose *via* the “copy and paste” mechanism in response to stress. Class II DNA transposons transpose *via* a “cut and paste” process in response to stress. In *Antirrhinum majus*, low temperature increases the excision frequency of the Ac/Ds type transposon Tam3 (Harrison and Fincham, 1964; Carpenter et al., 1987). Transposable elements are a response of the genome toward environmental challenges and play a critical role in gene regulation and evolution of the genome (McClintock, 1984; Slotkin and Martienssen, 2007; Fedoroff, 2012). It has been proposed that TEs activation in response to environmental stress could provide epigenetic variability which could contribute toward the greater adaptive capacity of plants under stress conditions (Mirouze and Paszkowski, 2011; Bucher et al., 2012; Ashapkin et al., 2020). The active DNA transposon mPing has been found to preferentially insert into 50 flanking regions of genes rather than exons in rice. Cold and salt stress encourages transcription of a subset of genes by inserting mPing in the promoter region (Naito et al., 2009; Wang et al., 2016).

It has been reported that epigenetic reprogramming has an important role in transposon silencing and reprogramming in germ cells of plants (Feng et al., 2010). In *Arabidopsis thaliana*, one egg cell and one central cell containing two nuclei are produced during the process of female gametogenesis, and several accessory cells are also produced. The egg cell fuses with one sperm cell during double fertilization process forming an embryo, and the second sperm cell fuses with the central cell leading to the formation of an endosperm (triploid). A helix hairpin DNA glycosylase, DEMETER (*DME*) causes hypomethylation in the endosperm by removing methylated cytosine residues. Demethylation *via DME* activates expression of transposons through the RNAi pathway that introduces transcripts of transposons and produces additional siRNAs guiding DNA methylation. The siRNAs expression in the endosperm development indicates genome imprinting, and siRNAs production guides DNA methylation in egg cell reinforcing transposons silencing in the germ cells. Transposons silencing occurs in the germ cells, but mild activity of transposons in endosperm have no major effects as the endosperm is not inherited to next generation (Hsieh et al., 2009b; Gehring et al., 2009; Ibarra et al., 2012; Ito 2013). Transposons activity in the embryo has been suppressed *via* the RNAi pathway having a major role in reprogramming of paternal genome in *Arabidopsis* (Han et al., 2019). It has also been reported that sRNAs lead to hypomethylation of vegetative cells when they are transported to the sperm cell *via* the cytoplasm of the pollen grains. This accumulates siRNAs and activates *de novo* remethylation through the RdDM pathway leading to transposons silencing in the gametes. Genes responsible for biogenesis of siRNA and transposons silencing are expressed at a very low concentration in pollen. But the *DDM1* gene is an exception, which is specifically expressed in the sperm cells of mature pollen. In the vegetative nucleus of wild-type, DNA demethylation and activation of transposons occur by the downregulation of the *DDM1* gene. This activation of transposons in vegetative cells also has no effect on the fitness of the species, as vegetative cells are not inherited to the next generation and have no contribution of genetic material, i.e. DNA, to the fertilized embryo. This demonstrates that epigenetic inheritance and transposon silencing are contributed through genome reprogramming guided *via* RNAs (Ito, 2013; Dziegielewski and Ziolkowski, 2021).

### miRNA Modifications

**FIGURE 2:**
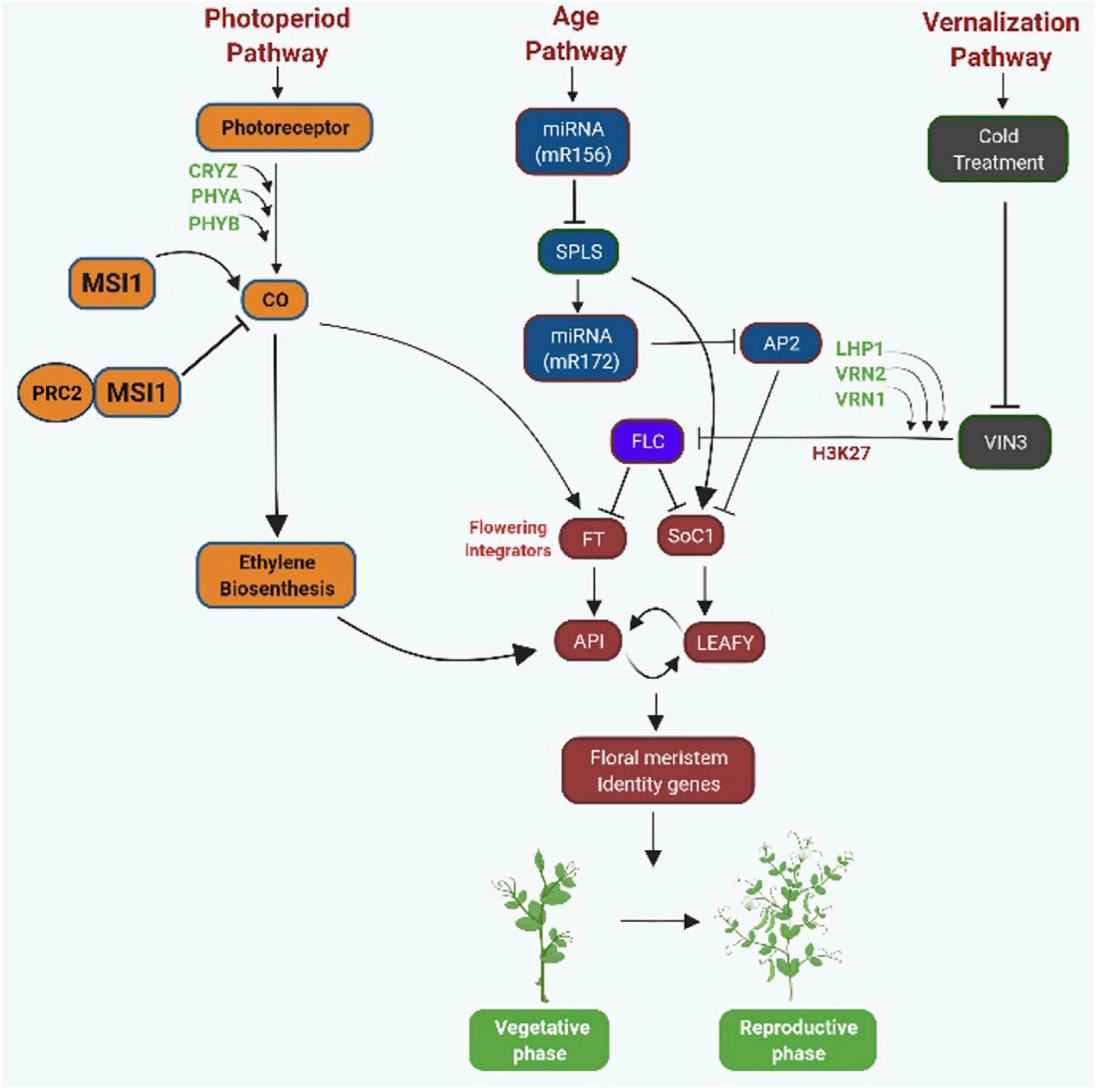
Role of epigenetic modifications in controlling flowering time in co-relation with biological pathways: flowering is induced by a number of molecular pathways that respond to external and internal signals. Flower integrator genes such as FT and SOC1 are regulated by flowering activators and repressors. In the photoperiodic pathway, chromatin modifications involve the well-conserved histone binding protein MULTICOPY SUPRESSORS OF IRA1 (MSI1)–like protein family. MSI1-like protein forms a complex with ubiquitous protein, i.e., POLYCOMB REPRESSIVE COMPLEX 2 in *Arabidopsis* and controls the switch to flowering. MSI1 acts in the normal expression of CO in long day (LD) plants. Reduced expression of CO in *msi1* mutants leads to FT and SOC1 repression. In age pathway, miR156 and miR172 acts as positive regulators of SOC1 gene. Vernalization leads to the expression of VIN3. VIN3 represses FLC transcription by binding with PcG protein (VRN1, VRN2, LHP1). PcG proteins epigenetically modify chromatin of FLC by trimethylation of H3K27.

miRNAs comprise 20–24 noncoding nucleotides that regulate gene expression after transcription and are also involved in the age pathway by regulating the time of flowering in plants by using RNA-directed DNA methylation (RdDM) (Matzke et al., 2001; Matzke et al., 2007; Pikaard, 2006; Teotia and Tang, 2015; Dziegielewski and Ziolkowski, 2021) (Figure 1). The miRNA molecules with the help of the RdDM pathway can bring about DNA methylation on a specific location (Teotia and Tang, 2015). Global gene expression analysis in *Arabidopsis thaliana* having genetic disorder in photoperiodic signaling pathway and system integrate genes suggesting the role of miRNAs in mediating the effects of floral induction (Schmid et al., 2003; Kinoshita and Richter, 2020). miR156 and miR172 are the two key miRNAs acting as the main elements in controlling the age pathways in plants by downregulating target genes and also effecting flowering time in many plant species (Figure 2). The level of miR156 increases during the vegetative stage of plants and decreases as plants proceed toward the reproductive stage, and at this stage, miR172 increases (Tanaka et al., 2011; Luo et al., 2013; Teotia and Tang, 2015). The expression of miRNAs is determined by environmental factors. miRNAs affect the expression of certain genes of plants when exposed to abiotic and biotic stress by frequently reprogramming genes involved in the developmental pathways (Covarrubias and Reyes, 2010; Hirayama and Shinozaki, 2010; Urano et al., 2010; Tiwari and Rajam, 2022) (Figure 1). Stress-inducible miRNAs and their expected targets have been discovered to be preserved in *Arabidopsis* (Sunkar and Zhu, 2004). It has been reported through global gene expression in rice plants that when exposed to stress conditions such as cold, drought, excessive salt, and ABA treatment, miRNAs modulate gene expression in the rice plants (Shen et al., 2010). Correlation between miRNA biogenesis mechanism proteins, response to stress, and flowering has been found in many mutant lines of *Arabidopsis*. For example, ABH1 and CBP20 encode cap-binding factors which are required for maturation of RNA (Papp et al., 2004). The *abh1* mutant exhibits ABA hypersensitivity, and the *cbp20* line exhibits both drought tolerance and ABA hypersensitivity (Hugouvieux et al., 2001; Kwak et al., 2005). In addition to the role of miRNA in stress responses, they are also important in controlling the flowering in *Arabidopsis* (Aukerman and Sakai, 2003; Chen and Li, 2004; Sunkar and Zhu, 2004). Long intronic noncoding RNA (COLDAIR) mediates interaction of H3K27me3 at FLC. This interaction of COLDAIR with FLOWERING LOCUS C (FLC) gene targets PRC2 to interact with FLC, resulting in FLC suppression during cold treatment, i.e., vernalization (Baulcombe and Dean, 2014). miRNA partially regulates the FL, and mutations in the miRNA biogenesis genes DCL1 and DCL3 cause delayed flowering in these mutant backgrounds due to overly high FLC expression (Schmitz et al., 2007) (Figure 1). Another mutant line HYPONASTIC LEAVES 1 (*HYL1*) was also found to show late flowering characteristics (Lu and Fedoroff, 2000). HYPONASTIC LEAVES 1 (HYL1) gene produces a protein that binds to double-stranded RNA (dsRNA) and mediates gene control *via* miRNA (Han et al., 2004). Scientists have reported that in addition to *hyl1* mutants, many *Arabidopsis* mutants in miRNA biogenesis machinery genes have phenotype related to ABA and salt hypersensitivity, i.e., SERRATE (SE) gene, DCL1 gene, HUA-ENHANCER 1 (HEN1) gene, and HASTY gene (Lu and Fedoroff, 2000; Han et al., 2004; Rasia et al., 2010; Zhang et al., 2018).

### Epigenetic Regulation in Plant Development and Morphogenesis

Stem cells are present in plant meristems and lead to the formation of all tissues and organs. The RdDM factor transcript levels are higher in tissues of meristem in *A. thaliana* than in cell expansion tissues, e. g., tissues of hypocotyl and differentiated leaves (Zhang et al., 2018). The DNA methylation level was found to be more in columella cells of root meristem because these cells are least condensed with pericentromeric chromatin. This allows more accessibility to RdDM factors. There have been no obvious reports of apparent meristem abnormalities in RdDM mutants in *A. thaliana*, but rice and maize mutants show severe developmental defects, indicating that these components play critical roles in meristem function (Zhu et al., 2012; Kawakatsu et al., 2017; Zhang et al., 2018). After the emergence of leaves from shoot apical meristem, many developmental genes get suppressed by the deposition of SET DOMAIN GROUP PROTEIN 711 (SDG711)–dependent H3K27me3 in rice. SDG711-dependent H3K27me3 synchronizes with DRM2-catalyzed non-CG DNA methylation in the gene body of *Oryza sativa*. SDG711 interacts physically with DRM2, and mutation in DRM2 decreases chromatin binding of SDG711 and deposition of H3K27me3 at repressed gene sites (Zhou et al., 2016; Zhang et al., 2018). During the growth of leaves in maize, DNA methyltransferases were found to be regulated variably resulting in distinct patterns of CG and CHG methylation in the division zone, transition, elongation, and mature zone reflecting the leaf’s spatial gradient of cells (Zhou et al., 2016). DNA methylation is required for pattern development in some leaf epidermal cells of *Arabidopsis thaliana*. DNA demethylation in *Arabidopsis thaliana* is dependent on genes of the ROS1 subfamily encoding 5-methylcytosine DNA glycosylases/lyases. DNA demethylation initiated *via* ROS1 leads to the expression of EPF2 gene. EPIDERMAL PATTERNING FACTOR 2 (EPF2) is a peptide ligand that inhibits stomatal development, resulting in an excess of stomatal lineage cells. Malfunction of ROS1 results in promoter hypermethylation and suppression of the gene encoding EPF2, resulting in stomatal cell development (Yamamuro et al., 2014; Zhou et al., 2016; Zhang et al., 2018). Similarly, the loss of function of H3K9 demethylase IBM1 (increase in BONSAI methylation 1) causes elevated H3K9me2 and CHG DNA methylation, as well as the suppression of three LRR RECEPTOR-LIKE SERINE/THREONINE-PROTEIN KINASE ERECTA family genes that encode EPF2 receptors, resulting in stomatal pattern formation problems as seen in ROS1 mutant plants. In *ros1* plants, the mutation in RdDM factors, H3K9 methyltransferase SUVH4, and CMT3 in *ibm1* plants can rescue the stomatal pattern formation resulting through abnormal epigenetic regulation because DNA methylation of EPF2 promoter and silencing of EPF2 depends on RNA-directed DNA methylation. Also, ROS1 acts against RdDM action leading to the expression of EPF2. This indicates that two DNA methylation-mediated mechanisms are responsible for regulating leaf epidermal cell patterning in *A. thaliana* (Table 1) (Candaele et al., 2014; Yamamuro et al., 2014; Wang et al., 2016; Zhou et al., 2016; Zhang et al., 2018). FLC, a MADS box transcriptional repressor that keeps *Arabidopsis* apices in the vegetative stage, is downregulated by vernalization. As a result, epigenetic alterations at the FLC locus hasten flowering (Kim et al., 2009).

**TABLE1:** Role of epigenetically induced modifications in trait control, development, and morphogenesis of different plant species.

Species	Epigenetic modification	Plant developmental responses	References
*Arabidopsis thaliana*	DNA demethylation	Stomatal development	Yamamuro et al. (2014)
	DNA methylation	Diseases resistance	Zhang et al. (2018)
	Histone modification	Growth and development	Czermin et al. (2002)
	Histone modification	Development of sporophyte and phase transition	Shen et al. (2021)
	DNA methylation	Response to pathogen pathways and floral induction	Gao et al. (2003)
	mi RNA	Enhanced plant phenotypic vigor	Mondal et al. (2020)
	miRNA	Regulation of trichome distribution	Xue et al. (2014)
	Histone methylation	Response to drought stress	Van Dijk et al. (2010)
	Histone methylation	Response to necrotrophic fungi	Berr et al. (2010)
	Histone methylation	Response to salt stress	Kim et al. (2010)
	Histone methylation	Response to cold stress	Pavangadkar et al. (2010)
*Aegilops tauschii*	DNA hypomethylation	Plant defense response against pathogens	Geng et al. (2019)
*Alternanthera philoxeroides*	DNA methylation	Maintains leaf and stem morphology	Gao et al. (2010)
*Antirrhinum majus*	DNA methylation	Stem elongation	Gourcilleau et al. (2019)
*Brassica rapa*	DNA methylation/histone modifications	Increased crop yield	Hauben et al. (2009)
	DNA hypomethylation	Plant defense response against pathogens	Kellenberger et al. (2016)
*Citrullus lanatus*	DNA hypomethylation	Plant defense response against pathogens	Sun et al. (2019)
*Elaeis guineensis*	DNA methylation	Somaclonal variations	Jaligot et al. (2000)
*Glycine max*	DNA methylation and histone modifications	Response to salt stress	Song et al. (2012)
*Ilex aquifolium*	DNA methylation	Leaf development	Herrera and Bazaga (2012)
*Lycopersicon esculentum*	Small RNA	Enhanced plant vigor	Kundariya et al. (2020)
*Nicotiana tabacum*	DNA hypomethylation	Plant defense response	Wang et al. (2018)
*Nicotiana tabacum*	DNA methylation	Aluminum and salt stress	Choi et al. (2009)
	DNA methylation	More efficient genetic transformation of plants.	Yamagishi and Kikuta (2021)
	DNA methylation	Regulation of nutritive value	Quadrana et al. (2014)
*Oryza sativa*	DNA methylation	Leaf development	Zhang et al. (2018)
	miRNA	Floral abnormalities	Zhu et al. (2009)
	miRNA	Leaf development	Xie et al. (2012)
	Histone methylation	Gene expression under drought stress	Zong et al. (2012)
	Histone methylation	Stem elongation	Chen et al. (2013)
	DNA hypomethylation	Plant defense response	Atighi et al. (2020)
	Histone methylation	Control of transposon activity	Cui et al. (2013)
*Panicum virgatum*	miRNA	Morphological alterations	Fu et al. (2012)
*Torenia fournieri*	miRNA	Plant growth	Shikata et al. (2012)
*Trifolium pratense*	DNA methylation	Defense against stress	Yang et al. (2020)
*Vitis vinifera*	DNA methylation	Response to medium-high temperatures in regenerated plants	Baranek et al. (2015)
	DNA methylation	Authentication of plant origin	Xie et al. (2017)
*Zea mays*	DNA methylation	Improved yield	Tani et al. (2005)
	DNA methylation	Adaptive evolution	Xu et al. (2020)
	DNA methylation	Prediction of key phenotypes	Regulski et al. (2013)
	DNA methylation	Phenotypic predictor, independent of genetic polymorphism data	Xu et al. (2019)
	DNA methylation	Controls cell division in maize leaves	Candaele et al. (2014)
	Histone modifications	Enhance plant resilience to stress	Forestan et al. (2019)
	Small RNA	Used as complementary biomarkers in crops	Seifert et al. (2018)
Sugar beet	DNA methylation	Tolerance to bolting	Trap-Gentil et al. (2011)
Rubber trees	DNA methylation	Tolerance against cold stress	Tang et al. (2018)

### Epigenetic Regulation Under Environmental Stress

DNA methylation suppresses gene expression under stressful conditions, allowing the plant to conserve energy and strength for survival (Thapa and Shrestha, 2020). During water scarcity, CAM plants show the transition from C3 photosynthetic cycle to CAM pathways, which increases their resilience. This is accompanied by an increase in genomic methylation and hypermethylation of satellite DNA. Hypermethylation response is used to synthesize chromatin structure, which controls the expression of several genes and helps the plants to withstand stressful conditions. Hypermethylation was also discovered when the root tip of pea plants was exposed to water scarcity conditions (Thapa and Shrestha, 2020). The vernalization process, which involves prolonged exposure to cold conditions, initiates flowering in some plant species and is a well-studied example of how cold causes epigenetic changes that affect flowering. Epigenetic regulator, NRPD1, a DNA-binding bromodomain-containing protein, AtGCN5-related GNAT family 5 (acetyltransferase 5) and histone deacetylase were upregulated in *Arabidopsis* (Lee et al., 2005). Low temperature has been linked to DNA demethylation in *Arabidopsis* and other plant species like *Zea mays* (Steward et al., 2002), *Antirrhinum majus*, and *Triticum aestivum* (Sherman and Talbert, 2002; Hashida et al., 2003; Hashida et al., 2003) (Table 1). The *Arabidopsis* VERNALIZATION INSENSITIVE 3 gene (VIN3), a chromatin-remodeling plant homeodomain (PHD) finger protein that increases acetylation levels, is induced by cold exposure. This protein is essential for FLC repression and flowering enhancement. Because FLC expression is not lowered by cold treatment, the mutant lines for VIN3 do not respond to vernalization and so remain in a vegetative state for longer durations (Sung and Amasino, 2004; Soppe et al., 2021) (Table 1). During vernalization, this complex attaches to VIN3 locus chromatin (Schonrock et al., 2006). In *Arabidopsis*, on the other hand, a decrease in H3K27me3 modifications within the histones of the cold-responsive gene COR15A and the GALACTINOL SYNTHASE gene ATGOLS3 results in enhanced gene expression (Taji et al., 2002; Kwon et al., 2009). Similarly, during dehydration stress, the plant trithorax factor (ATX1) (Alvarez-Venegas et al., 2003) tri-methylates Lys4 residues of histone H3 (H3K4me3), regulating floral organ development and altering expression of transcription factor WRKY70 (Alvarez-Venegas et al., 2007). ATX1 mutations result in severe flaws in floral architecture (Alvarez-Venegas et al., 2003).

**FIGURE 3:**
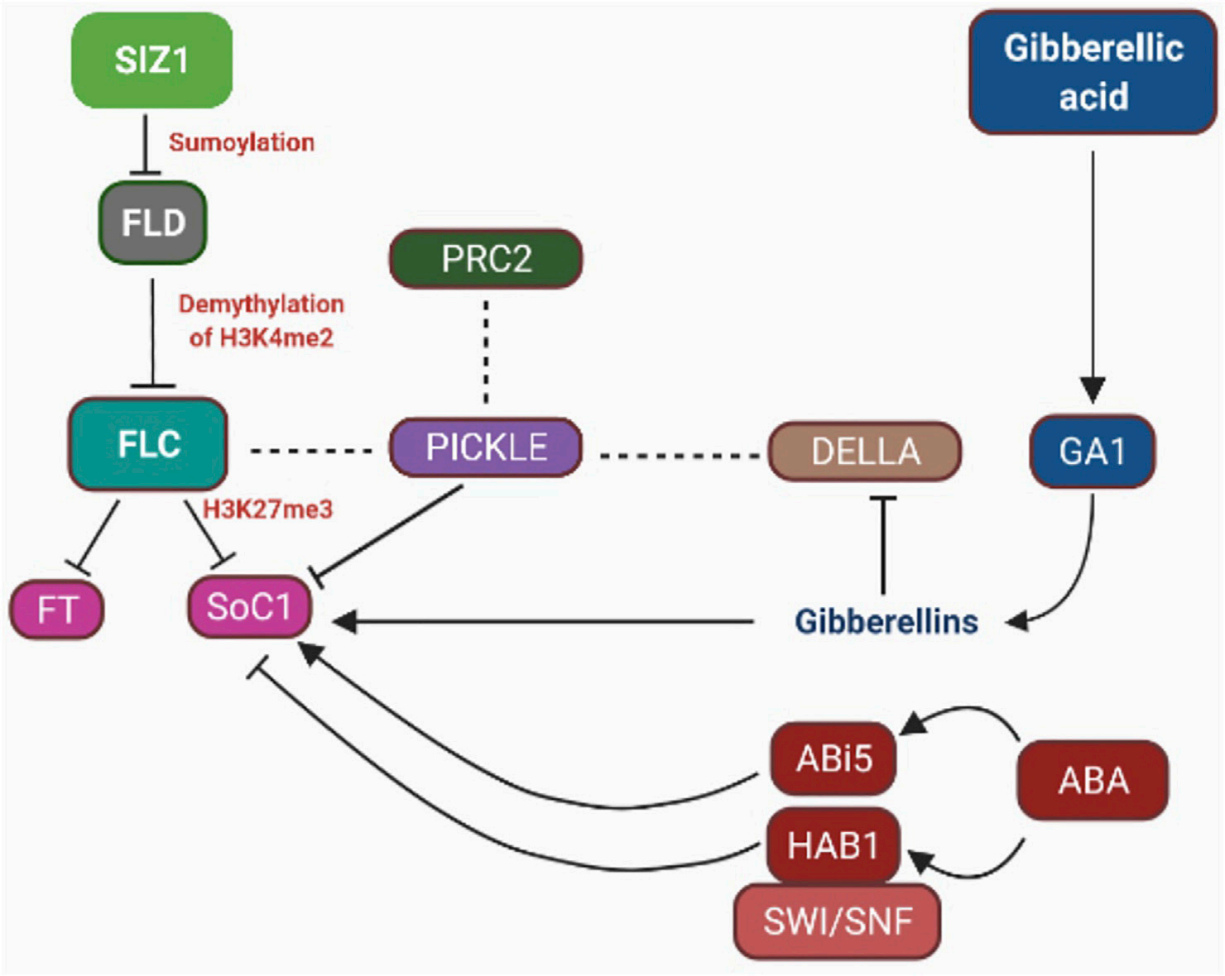
Epigenetic control on phytohormones: DELLA proteins are negative regulators of SOC1. DELLA proteins in combination with FLC interact with PICKLE (chromatin-remodeling protein) and PRC2 and repress the expression of SOC1. PRC2 regulates GA signaling by increasing H3K27me3-repressing histone protein. ABA INSENSITIVE MUTANT 5 overexpression delays flowering initiation by upregulating the expression of FLC. ABA HYPERSENSITIVE 1 (HAB1) is a negative regulator of flowering when combine with the chromatin-remodeling complex SWI/SNF. FLC is negatively regulated by lysine-specific demethylase 1–type histone demethylase (FLD). FLD causes demethylation of H3 histone (H3K4me3) in FLC. FLC downregulates SOC1 expression by trimethylation of lysine 27 on histone H3 protein *via* the formation of heterochromatic regions. SUMO E3 ligase (SIZ1) promotes expression of FLC by facilitating repression of FLD through sumoylation.

Various studies have reported that in *A. thaliana* and other plant species, including apples, *Pharbitis nil*, plant hormones such as auxin, cytokinin, Gibberellic acid, and abscisic acid interact to control flowering (Domagalska et al., 2010; Matsoukas, 2014). Salicylic acid (SA) is implicated in the control of CONSTANS, FLOWERING LOCUS C, FLOWERING LOCUS T, and MADS-box protein SOC1 transcription (Martinez et al., 2004). Interestingly, late-blooming phenotype of SA-deficient plants coincides with a 2- to 3-fold expression of FLC, lowering the FT levels in LD or SD circumstances as compared to wild-type plants. Furthermore, chromatin alterations are involved in the dynamic shift in the gene expression (Sun et al., 2014). For example, FLC and FT expression in *A. thaliana* is controlled epigenetically (Swiezewski et al., 2009; Ietswaart et al., 2012) (Figures 2, 3). It has been reported that under cold stress, Polycom Repressive Complex 2 (PRC2) is involved in silencing the FLC locus (floral repressor) through H3K27me3 (Yuan et al., 2016) (Figure 2). Also, some studies reported that the silencing of FLC successfully brought about through reducing H3K4me2 levels in FLC gene (Liu et al., 2010). PRC2 and Flowering Locus D (FLD) work in coordination to silence FLC (Shafiq et al., 2016; Campos-Rivero et al., 2017). Sumoylation/desumoylation action of the FLD gene can regulate acetylation/deacetylation of histones through the unspecified procedure. Histone demethylase is encoded by the FLD gene, thereby mediating H3K4me2 demethylation and facilitating H4 histone deacetylation in FLC chromatin (Jin et al., 2008). Exposure to various amounts of synthetic auxins results in epigenetic alterations that affect flower growth (Jaligot et al., 2000). The mantled phenotype of *Elaeis guineensis* Jacq (African oil palm) is characterized by anomalies in the development of flowers, leading to alteration in the auxin/cytokinin ratio (Table 2) (Eeuwens et al., 2002, Jaligot et al., 2011; Campos-Rivero et al., 2017). Mantled blooms were enhanced by applying a high amount of cytokinin (kinetin) and a low amount of auxin [1-naphthaleneacetic acid (NAA)], and there were less number of mantled flowers when a high amount of NAA and low amount of kinetin were applied (Eeuwens et al., 2002). Mantled phenotype resulted from DNA hypomethylation caused by kinetin, and the opposite phenotype of the plant resulted from DNA hypermethylation caused by NAA (Jaligot et al., 2000; Eeuwens et al., 2002, Jaligot et al., 2011). Pin-shaped inflorescence in *Arabidopsis thaliana* resulted from failed floral primordial initiation caused by Pin-formed mutant *pin-1*. This mutant diminishes the polar auxin transport thereby producing inflorescence devoid of flowers in *A. thaliana* (Okada et al., 1991). When IAA is given exogenously, it causes the production of flowers, which can be reversed (Reinhardt et al., 2000). Histone alterations have also been shown to have a role in transcriptional control of auxin target genes (Wu et al., 2015). mRNA accumulation increases in LFY and FILAMENTOUS FLOWER (FIL) when auxin is applied, resulting in initiation of the floral primordium. Auxin treatment leads to elevation of H3K9ac levels in LFY and FIL gene loci boosting flower primordial (Wu et al., 2015). In the absence of auxin, TOPLESS and HDA19 were reported to repress LFY and FIL loci through binding at their MP-sites, resulting in transcription inhibition of genes (Wu et al., 2015). Cytokinins play a major role in the division and differentiation of cells in the floral meristem (Schaller et al., 2015). Accumulation of cytokinins in *A. thaliana* meristem regulates the size of shoot apical meristem and the activity of cells in the shoot meristem. Cytokinin degradation is catalyzed by two cytokinin dehydrogenase enzymes CKX3 and CKX5 performing a regulatory function in the floral meristem of *A. thaliana* (Table 2) (Bartrina et al., 2011). In the central WUSCHEL (WUS) domain, CKX3 is expressed, and CKX5 expresses in the broad region of the apical meristem. Double mutants, i.e., *ckx3* and *ckx5*, lead to the formation of large inflorescence and flower meristem. Phenotype developed by these double mutants indicates that cytokinin signaling precisely identifies the niche of stem cells and retards the development of cells (Bartrina et al., 2011). Meijon et al. (2011) reported that cytokinins work in coordination with epigenetic modifications and regulate flowering processes in plants. Cytokinin dihydrozeatin riboside and isopentenyladenine end the dormancy period and influence flowering through the DNA methylation process. The levels of DNA methylation decrease before the initiation of flowers, but after the formation of floral organs, the levels of DNA methylation increase (Table 2) (Meijon et al., 2011). Scientists state that cytokinin is an epigenetic component whose function is to regulate gene expression during the transition from the vegetative to reproductive form by instigating demethylation. Abscisic acid (ABA) acts as a floral repressor in *A. thaliana* where externally applied ABA affects the blooming time (Wang et al., 2013). ABSCISIC ACID-INSENSITIVE MUTANT 5 (*AB15*) overexpression retards floral initiation through upregulation of expression of FLC (Wang et al., 2013). ABA INSENSITIVE 3 and ABA INSENSITIVE 5 genes also regulate flowering by encoding two transcription factors, i.e., basic leucine zipper (bZIP)–type and B3-type (Hauser et al., 2011). ABA HYPERSENSITIVE 1 (HAB1) protein is also involved in controlling flowering in *Arabidopsis* by interconnecting with SWI/SNF chromatin-remodeling complex during transcription induced by ABA (Figure 3). In response to cold stress, chromatin-remodeling affects the histone core proteins by increasing the concentration of ABA, which leads to an increased level of histone H3 acetylation (Saez et al., 2004). Abscisic acid (ABA) and ABA-responsive factors play an important role in the maintenance of bud dormancy in perennial plants such as the *Populus* species. The PtAB13 gene is a homolog of AB13 of *Arabidopsis*, and its overexpression and downregulation regulate seed dormancy *via* ABA signaling. It causes alterations in bud developmental processes and misregulates the expression of genes during the process of bud dormancy (Rohde et al., 2002; Ruttink et al., 2007; Rios et al., 2014) (Table 2). There is some evidence that epigenetic regulation and ET synthesis are linked (Zhou et al., 2005). It is well known that during cold stress, ET levels rise, which may be associated with vernalization processes (Figure 2) (Chu and Lee, 1989; Zhao et al., 2014), which are often regulated by DNA methylation or demethylation (Burn et al., 1993; Sherman and Talbert, 2002). Cold temperatures cause a rise in ET in *Arabidopsis*, which delay bloom; nevertheless, if the temperature is again appropriate, blooming is stimulated. Ethylene, on the other hand, has been shown to promote the expression of HDA6 and HDA19 (*Arabidopsis* HDACs) (Zhou et al., 2005). HDA6 upregulates FLC expression in *A. thaliana* (Wu et al., 2008) (Table 2). HAD19 links the hormone response to pathogen pathways and floral induction in *Brassica napus* through a similar epigenetic mechanism in response to variations in ethylene sensitivity. HAD19 has been shown to interact with bnKCP1 (a putative factor with a kinase-inducible domain), a cold-inducible factor that is highly expressed in flowers (Gao et al., 2003). The function of ethylene in the control of floral development and blossoming time has been demonstrated in roses (*Rosa hybrida* Samantha). ET is involved in the regulation of petal cell expansion during the opening of a rose flower (Table 2) (Ma et al., 2008). In roses, changes in miRNA expression in response to ethylene have been documented, with five miRNAs (miR156, miR164, miR166, miR139, and rhy-miRC1) demonstrating a strong link between ethylene and petal growth control (Table 2) (Pei et al., 2013). Plants use Jasmonic Acid (JA) and jasmonate molecules as signaling molecules in response to stress, such as mechanical or biotic injuries produced by ozone exposure, dehydration, or pathogen infection (Overmyer et al., 2000; Berger, 2002; Farmer et al., 2003; Loyola-Vargas et al., 2012). These, on the other hand, are involved in a variety of developmental processes, including nitrogen storage, fruit ripening, senescence, and blooming (Creelman and Mullet, 1995; Creelman and Mullet, 1997). The study of the epigenetic involvement in the control of JA during blooming has been less thorough than the study of the genetic role. However, significant breakthroughs have been made. In *Arabidopsis*, for example, HDA6, a HISTONE DEACETYLASE, is essential for JA response and flowering (Wu et al., 2008). This deacetylase, in collaboration with MET1, governs locus-directed heterochromatin silencing, potentially by recruiting MET1 to certain loci and therefore sets the groundwork for later non-CG methylation (To et al., 2011). HDA6 also physically binds with FLD (Yu et al., 2011; Liu et al., 2012), which contributes in the deacetylation of FLC chromatin and hence represses gene expression (He et al., 2003; Wu et al., 2008). This shows that HDA6 is involved in *Arabidopsis*' JA response and blooming (Figure 3).

**TABLE 2:** Summary of the role of epigenetic regulation along with phytohormone action in plant species.

Species	Trait	Hormone action	References
*Arabidopsis thaliana*	Production of flowers	Auxin (IAA)	Wu et al. (2015)
	Function in the floral meristem	Cytokinin dehydrogenase enzymes CKX3 and CKX5	Bartrina et al. (2011); Meijon et al. (2011)
	Flowering	ABA INSENSITIVE 3 and ABA INSENSITIVE 5 genes, ABA HYPERSENSITIVE 1 (HAB1) by interconnecting with SWI/SNF chromatin-remodeling complex	Saez et al. (2008); Bond et al. (2009)
	Delay in bloom	Rise in ethylene concentration	Achard et al. (2007)
	Flowering *via* FLC expression	HDA6 and HDA19 expression by ethylene	Zhou et al. (2005); Wu et al. (2008)
	Flowering	Jasmonic acid signaling *via* HDA6, a HISTONE DEACETYLASE	Liu et al. (2012); Yu et al. (2011)
*Brassica napus*	Response to pathogen pathways and floral induction	HAD19 expression *via* ethylene	Gao et al. (2003)
*Elaeis guineensis*	Development of flower	Auxin/cytokinin ratio	Eeuwens et al. (2002); Jaligot et al. (2011)
*Populus* species	Bud dormancy	Abscisic acid (ABA) and ABA-responsive factors, i.e., PtAB13	Rohde et al. (2002); Ruttink et al. (2007); Rios et al. (2014)
*Rosa hybrid*	Control of floral development and blossoming time	changes in miRNA expression in response to ethylene	Ma et al. (2008); Pei et al. (2013)

### Epigenetic Regulation in Response to Biological Virulence

In response to pathogen infection and symbiotic microbe colonization, plants show genome-wide DNA methylation alterations (Xiao et al., 2021). The first evidence of epigenetic regulations in response to biotic factors was reported to control virulence *via* posttranscriptional gene silencing (PTGS). Transcriptional gene silencing (TGS) is another permanent defense mechanism against DNA viruses *via* RdDM (Figure 1). It has been reported that virus infection in *Arabidopsis* is controlled *via* m^6^A-specific methylation of the RNA genome in the alfalfa mosaic virus (AMV). ALKBH9B, an *Arabidopsis* protein having demethylase activity, removes m^6^A from ssRNA molecules and accumulates in the cytoplasm of the siRNA bodies. This process suggests the role of m^6^A demethylase in mRNA silencing (Ramirez-Prado et al., 2018; Martinez-Perez et al., 2017; Ashapkin et al., 2020) (Table 1). The demethylase DME is required for nodulation in *Medicago truncatula*. Several hundred genomic sites, including a small proportion of nodule-specific symbiosis genes, are variably methylated during nodule growth. In cyst nematode–infected soybean and *A. thaliana* roots, widespread DNA hypomethylation was detected. Differential DNA methylation in *A. thaliana* has been produced by *Pseudomonas syringae* pv. tomato str. DC3000 (Pst DC3000) (Dowen et al., 2012; Sahu et al., 2013). Differentially methylated cytosines have been found in CG and CHH contexts in gene-rich areas, specifically at the 5′ and 3′ ends of protein-coding genes. Also, Pst DC3000–responsive DNA methylation correlates negatively with neighboring gene expression levels across the genome, indicating that at the gene borders, DNA methylation is regulated dynamically and may contribute to differential gene expression in response to pathogens. In cucumber leaves and pollen grains, DNA methylation is caused by plant pathogenic ncRNAs promoter areas and transcriptional activation of some ribosomal RNA (rRNA) genes, leading in an abundance of short RNAs produced from rRNA. In *A. thaliana*, external application with pathogenic resistance phytohormone, i.e., salicylic acid, resulted in megabase-scale DNA hypomethylation in pericentromeric areas. DNA methylation has been proved to be a protective mechanism against unwanted transposition and a defense system against endonuclease digestion (Yaish, 2013). After infection with the virus, genomic methylation increases, and gene methylation related to resistance decreases. An increase in methylation promotes stability of the genome when plants are attacked by a virus, but genetic recombination is caused by a decrease in gene methylation levels and ultimately new genes produced help in resistance against pathogens (Engler et al., 1993).

Plant susceptibility to certain infectious pathogens can be altered by mutations in DNA methylation or demethylation regulators (Yu et al., 2013; Zhang et al., 2018). Plant tolerance to the biotrophic fungus *Hyaloperonospora arabidopsidis* is similarly increased in DNA hypomethylation mutants like *nrpe1* and diminished in DNA hypermethylation mutants like *ros1* (Sanchez et al., 2016; Zhang et al., 2018). POL V mutations lower susceptibility to the fungal necrotrophic pathogens *Botrytis cinerea* and *Plectosphaerella cucumerina*, in addition to enhancing resistance to biotrophic pathogens. An *nrpd1* (POL IV) mutant does not have altered resistance to Pst DC3000 or fungal infections, unlike POL V mutants, indicating that POL V can regulate plant immune responses independently of canonical RdDM (Lopez et al., 2011; Zhang et al., 2018). Plants with the AGO4 mutant alleles *ago4-1* and *ago4-2*, on the other hand, showed higher vulnerability to Pst DC3000, suggesting that AGO4 plays a distinct role in plant disease resistance compared to the other RdDM components. The DNA demethylase triple mutant *ros1–dml2–dml3* and wild-type *A. thaliana* were compared, and it was discovered that DNA hypermethylation occurred more frequently in the mutant at regions flanking the gene body, such as upstream promoter regions and three untranslated regions. In the *ros1–dml2–dml3* plants, over 200 genes are repressed, a large number of which have known or potential involvement in biotic stress response and are enriched with tiny transposons in their promoters. The *ros1–dml2–dml3* mutant is more susceptible to the fungal disease *Fusarium oxysporum*, which supports this theory (Zhang et al., 2018) (Table 1).

### Epigenetic Regulation in Plant Reproduction and Meiosis

Epigenetic modifications display a significant contribution in the position and rate of crossovers; however, the mechanism of the molecular crossovers remains to be fully studied (Wibowo et al., 2016). The highly coordinated gene expression within the germ cells requires epigenetic reprogramming. Epigenetically induced molecular pathways play a vital role in essential chemical and physiological processes during plant meiosis. The transmission of epi-alleles produced in response to environmental pressures raises issues about how agronomic treatments and environmental circumstances may influence the expression of critical crop characteristics that are evaluated in particular genetic improvement techniques. Indeed, from the standpoint of agricultural genetic improvement, understanding the epigenetic regulation of plant reproduction and meiosis is of great importance.

Meiotic siRNAs play an important role in epigenetic control of meiotic chromosome condensation, with essential implications for crop genetic improvement. In maize, OUTER CELL LAYER 4 (OCL4) encodes a transcription factor HD-ZIP IV which is required for the biogenesis of small RNAs and the other 21-nt phasiRNA (Zhai et al., 2015). This transcription factor also induces the synthesis of other proteins belonging to pentatricopeptide repeat (PPR) proteins, NB-LRR, and MYB families in other species (Howell et al., 2007; Zhu et al., 2012). It has been established that the presence of the histone variation H2A.Z is favorably linked to the occurrence of crossovers (Shilo et al., 2015). Plant crossover hotspots are suppressed by DNA methylation and H3K9me2 (Yelina et al., 2015). In *Arabidopsis*, the loss of DNA methylation has also been demonstrated to change crossover distribution in a chromatin type–dependent way (Mirouze et al., 2012).

SPO11-1-oligonucleotides and SPO11 topoisomerase-like transesterases produce DNA double-strand breaks to generate a high-resolution method to profile meiotic double-strand break patterns genome-wide. SPO11-1-oligonucleotides have been mapped in the *Arabidopsis* genome, and their role in regulating chromatin, DNA, and crossover frequency has been studied (Choi et al., 2018). The identification and mapping of these short DNA sequences in crop genomes will be important to discover epigenetic markers associated with key epigenetic modulators. Another important gene involved in the epigenetic modulation of plant reproduction is DECREASE IN DNA METHYLATION 1 (DDM1). DDM1 has ATPase activity that controls DNA methylation linked to crossover occurrence (Castiglione et al., 2002; Higo et al., 2012).

### Male and Female Gametophyte Development

In plants, the primary germ cells do not directly enter spermatogenesis and oogenesis. Instead, the pollen mother cells (PMCs) in the anthers and the megaspore mother cells (MMCs) in the ovaries are generated in the floral meristem as a consequence of two meiotic divisions followed by a series of mitotic divisions, resulting in haploid male and female gametophytes, respectively (Manning et al., 2006). The male and female gametophytes are good models for investigating cell polarity, morphogenesis, and epigenetic regulation of cell development and specialization, and signaling pathways in angiosperms, despite their modest size and limited number of cells. Microsporogenesis and microgametogenesis are two phases of pollen formation that take place in the anthers. The sporogenous layer of the anther produces diploid microsporocytes, or PMCs. PMCs produce a tetrad of four haploid cells after two meiotic divisions. The tetrads then split up into individual microspores. Following that, two mitotic divisions occur: the first produces a big vegetative cell and a smaller generative cell, followed by the generative cell division, which produces two sperm cells, while the vegetative cell does not divide (Brownfield et al., 2009).

Epigenetic alterations are critical in the development of both male and female gametophytes, as well as in fertilization (Ingouff et al., 2017). It was discovered in *Arabidopsis* that the methylation level of PMCs in a normal environment (CG and CHG) was greater than in an adverse situation (CHH) (Kumar and Mohapatra, 2021). Symmetric methylation is generally seen in transposable elements, whereas asymmetric hypermethylation is mostly found in protein-coding genes. Increased methylation in a symmetric situation is believed to facilitate TE activity suppression, which guarantees genome stability before and throughout meiosis. Inactivation of methylation in an asymmetric setting, on the other hand, increases the activation of genes required for sperm cell development and conception. A substantial remodeling of chromatin occurs during PMC maturation, in addition to DNA methylation, promoting the start of meiosis.

The transition from the mitotic to meiotic phase is accompanied by a decrease in restrictive chromatin (H3K27me1 and H3K27me3) and an increase in permissive chromatin (H3K4me3) (Borg et al., 2020). The vegetative cell becomes roundish during meiosis and asymmetric during mitotic division of the haploid microspore. It has more methylation in the CHH areas, but it loses centromere-specific histone H3 (CENH3) due to decondensation of pericentromeric heterochromatin, local hypomethylation due to DME/ROS1 demethylases, and transposable element activation (Calarco et al., 2012).

During mitotic and meiotic cell division, the centromeric histone H3 (CENH3) variation is critical for the assembly and function of kinetochores. The inclusion of CENH3 into centromeric nucleosomes is the first step in kinetochore formation. The amount of CENH3 deposited on the centromeres changes depending on the stage of the cell cycle. CENH3 is also required for vegetative cell division and the removal of additional DNA (Lermontova et al., 2015). Hypomethylation of TEs leads in the production of 21–22 nt siRNAs, which are transferred to sperm cells and used by RdDM methylation to repress their TEs (Slotkin et al., 2009). During the whole time of pollen generation and development, whole-genome cell-specific methylation profiling indicated a high degree of CG and CHG methylation in the DNA of microspores, sperm, and vegetative cells.

During the development of a megasporocyte, DNA methylation level remains unchanged in context to CG, while it decreases temporarily in the context of CHH (Ingouff et al., 2017). The MMC, as well as the functional megaspore, are specified and differentiated through intercellular interactions mediated by mobile trans activating siRNAs (tasiRNAs), which are produced in the nucellus' surrounding cells and transported to the MMC, where they implement transcriptional and translational silencing (Baulcombe and Dean, 2014). The AGO9, RDR6, and SDS3 (a suppressor of genetic silencing 3) enzymes have been demonstrated to control the generation of such siRNAs in *Arabidopsis* (Olmedo-Movolif et al., 2010). The onset of mega gametogenesis in *Arabidopsis* nucellus is hampered when AGO5 expression is disrupted. Throughout mega gametogenesis, methylation in the CG and CHH contexts stays constant. CG methylation inside genes and transposons of the central cell of the embryo sac was lower than that of sperm cells, as shown in *Arabidopsis* (Park et al., 2016). This suggests that even before fertilization, the potential transcription of male genes is repressed. Mobile noncoding tasiRNAs regulate epigenetic control during gametogenesis in the embryo sac, thus siRNAs from the central cell penetrate the egg cell and decrease transposable element activity.

### Fertilization and Embryogenesis

In both the embryo and endosperm, fertilization eliminates CHH hypomethylation of the paternal genome (Ibarra et al., 2012). Remethylation of the paternal DNA is most likely mediated by maternal siRNAs. One rationale for the epigenetic suppression of the male genome during early embryogenesis might include maternal regulation of embryo and endosperm size, as well as detection of self-pollen, which is important in interspecific crosses (Creasey et al., 2014). For appropriate embryo development, proper and consistent methylation of the dividing egg cell DNA is critical. In comparison to mature embryos, young embryos and endosperm tissues are hypomethylated, which represents the high transcriptional activity of genes in the developing embryo and the preparation for the embryo’s dormancy (Bouyer et al., 2017; Kawakatsu et al., 2017).

The levels of DNA methylation are strictly controlled in distinct tissues and cell types throughout the life cycle of a plant. In comparison to the embryos, endosperms of *Oryza sativa* and *A. thaliana* show worldwide DNA hypomethylation. It is caused by DME-dependent active demethylation in the central cell before fertilization in *A. thaliana* (Gehring et al., 2009; Hseih et al., 2009a; Ibarra et al., 2012). MET1 transcriptional repression also occurs during female gametogenesis, but it is unable to play a role in demethylation. Because genome-wide CG hypomethylation was not found in wild-type endosperm, DNA methylation is recovered in the dme mutant endosperm (Ibarra et al., 2012; Park et al., 2016). The vegetative cells experience DME-dependent DNA demethylation, which is accompanied by significant DDM1 downregulation (Table 3) (Slotkin et al., 2009; Ibarra et al., 2012). As a result, demethylated and de-silenced transposons create siRNAs. The siRNAs reach the vegetative cell after passing through the sperm cells, fortifying the RdDM pathway. POL V and DRM2 but not POL IV of *A. thaliana* was found in egg cells. These are the requirements for generation of siRNA through the conventional RdDM pathway. Therefore, transposon siRNAs accumulation in the sperm cells may enhance transposon silencing following fertilization of egg cells. During seed development, there is a rise in global levels of CHH methylation, and during seed germination, the levels fall due to passive demethylation.

**TABLE 3:** Role of epigenetically induced modifications in reproduction of different plant species.

Plant species	Plant characteristic/s	Epigenetic mechanism	References
*Arabidopsis thaliana*	Flowering phenology	DNA methylation	Borrdorf et al. (2010); Zhang et al. (2013)
	Control of flowering time	Chromatin modifications	He (2009)
	Control of flowering time	Histone methylation	Deal et al. (2007); Tamada et al. (2009)
	Control of flowering time	Epigenetic repression through vernalization	Sheldon et al., 2008
	Control of flowering and senescence	Histone methylation *via* Jasmonate signaling	Wu et al. (2008)
	Temperature response and flowering time	Histone modification	He et al. (2011)
	Increased stamen and carpel numbers	Cytosine methylation	Jacobsen and Meyerowitz (1997)
	Double-fertilization	DNA demethylation	Gehring et al. (2009); Hsieh et al. (2009b); Ibarra et al. (2012)
	Seed dormancy	DNA demethylation	Bouyer et al. (2017); Zhang et al. (2018)
	Fertilization of egg cells	Transposon silencing *via* RdDM pathway	Bouyer et al. (2017); Zhang et al. (2018)
	Transcriptional gene silencing	DNA methylation *via* RdDM pathway	Xie et al. (2012)
	Phenotype with late flowering	DNA methylation	Ghoshal and Gardiner, (2021)
	Transcriptional gene silencing (TGS)	DNA methylation	Ramirez-Prado et al. (2018), Ashapkin et al. (2020)
	Mega-gametogenesis and endosperm development	Histone modification	Shen et al. (2021)
	Flowering, signaling of hormones and circadian clock control	Histone modification	Zheng and Gehring (2019); Sanchez et al. (2020)
*Brachypodium distachyon*	Control of flowering time	miRNA	Wu et al. (2013)
*Gossypium barbadense*	Flowering time and seed dormancy	DNA methylation	Song et al. (2017)
*Linaria vulgaris*	Peloric flowers with abnormal actinomorphic flowers	Cytosine methylation	Cubas et al. (1999)
*Lycopersicon esculentum*	aberrant flowers and parthenocarpic fruits	Histone deacetylation	Ampomah-Dwamena et al. (2002)
	Fruit ripening	DNA methylation	Zhong et al. (2013)
*Marchantia polymorpha*	Spermatogenesis	DNA methylation	Kumar and Mohapatra, (2021)
*Oryza sativa*	Flowering time	Histone modification	Sun et al. (2014)
	Flowering and reproduction	Histone modification, DNA methylation	Shi et al. (2015)
*Populus* Spp.	Bud dormancy	DNA methylation	Rohde et al. (2002)
*Prunus mume*	Bud dormancy	DNA methylation	Zhong et al. (2013)
*Pyrus pyrifolia*	Bud dormancy	DNA methylation	Liu et al. (2012)
*Rubus idaeus*	Bud dormancy	DNA methylation	Mazzitelli et al. (2007)
*Sinningia speciosa*	Flowering time control	miRNA	Li et al. (2013)
*Solanum lycopersicum*	Fruit ripening	DNA methylation	Manning et al. (2006)
*Solanum lycopersicum*	Fruit ripening	Histone deacetylation	Zhao et al. (2014)
*Solanum ruiz-lealii*	Flower abnormalities	DNA methylation	Marfil et al. (2009)
*Spinacia oleracea*	Artificial induction of flowering	DNA methylation	Cheng et al. (2019)

During seed germination, however, the metabolic and, as a result, transcriptional-genetic activity of the embryonic tissues increases again, accompanied by a reduction in methylation in the CHH context, which is linked to the activation of protein-coding gene expression (Papareddy et al., 2020). The epigenetic “memory” associated with the histone marks is removed and is not transferred to the following generations of cells because the histones inherited from the egg and sperm are not reproduced in the cells of the embryo but are synthesized afresh (Papareddy et al., 2020). Thus, embryogenesis comes after the meiosis checkpoint or clearing box, which eliminates the maternal sporophyte’s epigenetic markers from the DNA. Endosperm methylation is substantially lower than that of the embryo, reflecting its strong transcriptional and metabolic activity.

At the same time, paternal genomes (i.e., genomes transported by sperm cells into the egg cell and the central cell of the embryo sac) are more methylated than maternal genomes (Heish et al., 2009a). Endosperm demethylation appears to be necessary to diminish transposable element activity *via* the production of siRNAs, which are transported into neighboring embryo cells and methylate the terminal sections of transposons by RNA-directed DNA methylation, thus inactivating them (Ingouff et al., 2017).

### Fruit Ripening

About 1% of the DNA methylation at cytosine–phosphate–guanine sites in the fruit pericarp of tomato gets changed during fruit development (Lang et al., 2017; Zhang et al., 2018). Many fruit-ripening genes have active DNA demethylation because their promoter regions contain binding sites for RIPENING-INHIBITOR (RIN), a prominent ripening transcription factor. Most known ripening genes whose expressions were adversely linked with promoter DNA methylation levels had confirmed RIN binding to target promoters. Premature ripening of tomato fruits was induced by treatment with a chemical inhibitor of DNA methylation, which caused promoter hypomethylation and expression of the gene encoding COLORLESS NON-RIPENING (CNR), which is a critical RIN-targeted gene for fruit ripening (Gao et al., 2010; Liu et al., 2015; Lang et al., 2017). The gradual DNA demethylation that occur during fruit ripening in *Solanum lycopersicum* is mediated by DML2. The expression of DNA demethylase DME-LIKE 2 (DML2) increases rapidly in ripening fruits. In *S. lycopersicum*, DML2 targets both ripening-induced and ripening-repressed genes, implying that active DNA demethylation is necessary for both ripening-induced gene activation and ripening-repressed gene suppression (Telias et al., 2011; El-Sharkaw et al., 2015; Daccord et al., 2017; Zhang et al., 2018) (Table 3).

### Seed Dormancy

In perennial plants, epigenetic modifications regulate seasonal dormancy cycles. Meristem and bud growth are controlled by photoperiod, temperature, etc. Transcriptomic studies have revealed that bud dormancy events in plants such as *Populus* spp., *Rubus idaeus*, *Euphorbia esula*, *Prunus mume*, *Vitis* spp., *Prunus persica*, and many other perennial plant species have been triggered through changes in their gene expression. These changes affect regulation of cell cycle, perception of light, signaling of hormones, and response to stress (Rios et al., 2014). Characterization and identification of nondormant mutants of perennial plants, such as *evergrowing* (*evg*) mutant of *Prunus persica*, contributed toward increasing molecular work at the level of genes, thereby renewing the field of dormancy. Deletion in tandemly repeated sequences of MADS-box genes, i.e., DORMANCY-ASSOCIATED MADS-box gene (DAM1-6) leads to nondormant phenotype of *evg* mutant. DAM genes are expressed in buds and get affected by photoperiod and chilling temperatures, thereby affecting the developmental stages of plants (Lloret et al., 2021). The concentration of DAM genes, i.e., DAM5 and DAM6, was found to be high in dormant buds, and after chilling treatment, the level fell and released the dormancy of buds (Yamane et al., 2011). In many other perennial species, such as *Rubus idaeus*, *Prunus mume*, *Pyrus pyrifolia*, and *Actinidia deliciosa*, DAM-like genes have been found to have dormancy-dependent expressions. In transgenic plants, the heterologous expression of these genes has regulatory roles in flowering and dormancy. In *Arabidopsis*, the expression of DAM1 and of SVP1 and SVP3 in *Actinidia deliciosa* led to delay in the time of flowering (Horvath et al., 2010; Wu et al., 2012) (Table 3).

## Conclusion and Future Prospects

Plants being sessile in nature are invariably affected by changing environmental conditions. However, they have the ability to adapt their biological processes according to the changing environments. They interact with their environment through consistent adjustments at the molecular levels. Epigenetic mechanisms contribute to these adjustments. These changes within the plant species modify the gene expression and help different plant species to withstand the extremes of different biotic or abiotic constrains. Such changes are induced due to DNA methylation, histone modification, e.g. acetylation/deacetylation, methylation/demethylation, ubiquitination, phosphorylation, and sRNA/modifications, which work in tandem with respect to environmental signals, transposon silencing, and hormone signaling to control the expression of genes in plants. These alterations help to maintain the survival of plants and maximize the chances of sexual reproduction under stress conditions. The role of DNA methylation in plant improvement is the preferred mechanism to investigate gene function and manipulate plants for creating novel varieties having capability to survive under stress conditions. Therefore, improved knowledge of epigenetic mechanisms *via* thorough study at the molecular level will be helpful. FLC controls flowering time in *Arabidopsis* that involves many genes in FLC expression *via* chromatin modifications. Further studies have to be undertaken to know how these diverse epigenetic modifications interact with one another to regulate the expression of FLC. CRISPR-based systems can be useful in altering the expression of genes for research applications as well as crop improvement efforts. The generation of genetically viable agricultural varieties that can endure a warmer world relies heavily on information generated from the plants' epigenomic profiles subjected to environmental challenges. Understanding the dynamic management of histone methylation and how histone methylation controls plant growth would be expanded through biochemical and genetic studies. Identification of intrinsic histone demethylases in plants, particularly lysine and/or arginine demethylases, and elucidation of their functions in regulating plant development and genome stability would require biochemical and genetic evidence. The roles of regulation of H3K27me3 in plants are limited and more in-depth knowledge will enable new researchers to enhance productivity of crops under limiting environmental conditions.
